# A hypothesis study on bionic active noise reduction of auditory organs

**DOI:** 10.1186/s40779-018-0155-8

**Published:** 2018-02-26

**Authors:** Qing-Qing Jiang, Ning Yu, Shi-Ming Yang

**Affiliations:** Department of Otolaryngology Head and Neck Surgery, Institute of Otolaryngology, Chinese PLA General Hospital; Key Laboratory of Hearing Impairment Science, Ministry of Education; Beijing Key Laboratory of Hearing Impairment Prevention and Treatment, Beijing, 100853 China

**Keywords:** Active, Noise reduction, Bionic, Hearing loss

## Abstract

**Background:**

Noise exposure can lead to hearing loss and multiple system dysfunctions. As various forms of noise exist in our living environments, and our auditory organs are very sensitive to acoustic stimuli, it is a challenge to protect our hearing system in certain noisy environments.

**Presentation of the hypothesis:**

Herein, we propose that our hearing organ could serve as a noise eliminator for high intensity noise and enhance acoustic signal processing abilities by increasing the signal-noise ratio. For suprathreshold signals, the hearing system is capable of regulating the middle ear muscles and other structures to actively suppress the sound level to a safe range.

**Testing the hypothesis:**

To test our hypothesis, both mathematic model analyses and animal model studies are needed. Based on a digital 3D reconstructed model, every structure in the auditory system can be analyzed and tested for its contribution to the process of noise reduction. Products manufactured by this bionic method could be used and verified in animal models and volunteers.

**Implications:**

By mimicking the noise-reduction effect of the sophisticated structures in the hearing system, we may be able to provide a model that establishes a new active-sound-suppression mode. This innovative method may overcome the limited capabilities of current noise protection options and become a promising possibility for noise prevention.

## Background

Various types of noise have emerged and increased in our surroundings, e.g., traffic noise, industrial noise, construction noise and loud noise from civilian lives. Noise exposure can lead to not only hearing problem but also multiple system dysfunctions [1–5]. Protective devices such as earplugs and earmuffs have been developed to alleviate insults to recipients. However, this “passive protection” method has only a limited effect, and the efficacy mainly depends on training and proper use [6, 7].

The traditional method for preventing noise-induced damage is to block noise transmission with walls, sound-absorbent materials or sound-deadening materials. However, the most fundamental method is to decrease the sound energy generated from the sound source. The recent concept of “noise canceling” is based on the generation of a reverse signal by a digital signal processing system [8, 9]. This active method offers a new opportunity for noise prevention, but its restricted effect on high-frequency noise has limited its promotion. So, an efficient system that could actively decrease noise generation is an urgent need for both social development and the military industry.

Herein, we are attempting to propose a brand new hypothesis named “bionic active noise reduction” by presenting the innate structures in the auditory system that may contribute to noise suppression. The novelty of our hypothesis is the use of a bionic method to replicate the relevant hearing structures and their noise-reduction functions to achieve better efficiency in noise protection. The application of such active noise-reduction structures may possibly overcome the current limitations in active noise prevention. Once this hypothesis can be verified, it may have significant implications for noise protection in social medical services and national economies.

## Presentation of the hypothesis

i. Noise reduction in the auditory system

Our acoustic apparatus has evolved into an elaborate organ throughout human history. Within a response range of 20 Hz to 20,000 Hz, it is required to be not only sensitive but also durable to protect itself from injury. Researchers have focused on its precise signal processing ability, but its self-protective noise-reduction mechanism has never been noticed.

In normal physiological conditions, the internal noise of our body, such as the sounds of breathing, blood flow, muscle contractions and joint movements, are suppressed by our internal “sound-reduction system”. This system addresses the internal noise made within oneself and prevents it from influencing external sound processing. Vascular murmurs, created by hemodynamic changes in blood flow, could lead to audible pulses, known as pulsatile tinnitus [10–12].

More importantly, for external noise, we think that this innate “sound-reduction system” could actively process external sounds to minimize excessive energy. Sound signals are amplified by the signal amplification system to maintain its sensibility and clarity Furthermore, for suprathreshold signals, the auditory system could actively suppress signals to prevent injury.

For patients with hearing loss, both the ability to sense signals and the ability to reduce noise are decreased. Simply amplifying the signal with a hearing aid cannot mimic the denoising effect of the normal system and makes the amplified sound unbearable. The excessive energy has been dissipated via the middle and inner ear in a functioning auditory system, and if it was not, which indicates a loss of the active noise-reduction function, hearing damage would occur. This process prevents disturbances from our internal physical noise and protects our vulnerable hearing system from being damaged by external intense noise.

ii. Features of the active noise-reduction system

- Noise-blocking and reducing effect of the Eustachian tube (ET)

The ET is a key passage between the nasal cavity and the middle ear. The pharyngeal orifice of the ET works as a valve, which when closed prevents internal noise, for example, self-talking and breathing, from being transmitting to the tympanic cavity. The dysfunction of the ET invariably leads to middle ear problems, such as aural fullness, pain, tinnitus and otitis media [13–15]. If the air from breathing flows through the abnormally open ET, the sounds of breathing will be heard and even mask external signal perceptions.

The external 1/3 of the ET, the tympanic section, is an open, funnel-shaped tube covered with mucosal folds [16]. These features are somewhat similar to a muffler. We suppose that these features could help dissipate the vibrational energy of the tympanic membrane and round window. This will also assist in the reduction of acoustic signals in the middle ear and balance the middle ear pressure. For intense sounds, the ET could act as an important mechanism to reduce pressure and energy. This can be proven by the fact that opening the mouth can protect the tympanic membrane from loud noise exposure [17]. In short, the ET plays an important role in the noise-reduction system and functions to both block noise from entering the inner ear and dissipate the energy in the middle ear.

b) Function of middle ear skeletal muscles in the protection from noise-induced injury

The middle ear muscles, the tensor tympani and stapedius muscles, also have important functions in noise protection. The stapedius reflex is a significant protective reflex when the ear is stimulated by intense sounds. This mechanism reduces the excessive energy that passes into the inner ear. The amplitude of the stapedius reflex is decreased in patients with noise-induced hearing abnormities even when their thresholds are normal at 1000 Hz, which confirms that their noise-reduction system is affected [18]. A researcher showed that the stapedius reflex has an important function in decreasing internal sound in order to perceive external signals better [19]. This has a positive effect on language perception under noisy conditions. The contraction of the tensor tympani could prevent the rupture of the tympanic membrane and damage to the inner ear. It is sensitive to mechanical stimuli and protects the hearing system from loud sounds.

The reflexes of both the stapedius and tensor tympani are important innate mechanisms that prevent noise damage by effectively decreasing the intense noise rushing into the inner ear.

c) Function of inner-ear-related structures in noise protection
  The oval window, blocked by the stapes footplate and ligament, transfers sound energy from middle ear to the inner ear. The structure of the footplate and ligament ensures the efficient and precise conductive movement of the oval window. It is notable that the surrounding ligament could limit the vibration of the oval window in response to loud sound stimuli within an appropriate range. This prevents hair cell injury caused by excessive basilar membrane movement.
  The moderate transmission of sound wave energy in the inner ear from liquid (perilymph) to solid (basilar membrane) can largely avoid direct mechanical injury to hair cells. On the other hand, the existence of endolymph and perilymph could facilitate the later attenuation of sound when waves dissipate in the fluid. The vibration of the perilymph causes the round window to vibrate, and energy is buffered and reduced through vibration and deformation.
  The endolymph and perilymph are connected with the endolymphatic sac and the cochlear aqueduct. We presume that these structures also facilitate the energy dissipation. When hydrolabyrinth and aqueduct obstructions emerge, lymphatic discharge abnormities will cause vestibular dysfunction and hearing loss, such as Meniere’s disease. Large vestibular aqueduct dysfunction also leads to severe hearing loss.
  The noise-reduction system we proposed involves the mentioned structures in both the middle ear and the inner ear, and it works as a cooperative system to protect the hearing system from intense noise injury. When the system fails to limit the sound signal within an appropriate range, inner ear damage occurs and may eventually develop into noise-induced hearing loss.

iii. Progress in research on the pathogenesis and therapy of noise-induced hearing loss

- Pathogenesis of noise-induced hearing loss damage

Understanding the mechanisms of noise-induced hearing loss is still a challenge in otolaryngology. Nordmann proposed the theory of cilium damage in the year 2000 [20]. Our group discovered that hair cells could maintain normal cellular function for 2 weeks after cilium was impaired [21]. The first 2 weeks became the optimal timeframe for hearing recovery. This work first proved the self-healing ability of tip-links after hair cell cilium damage [22]. We also discovered a cascading effect after hearing injury and proposed a four-stage inner ear pathology theory. We further established the basic strategy of hair cell regeneration and gene therapy after hearing loss [23]. The mechanism of neurotrophic factors in the improvement of hearing is the utilization of the self-healing ability of tip-links.

b) Progress in the therapy of noise-induced hearing loss

1. Surgical therapy of noise-induced hearing loss promoted the development of otology

Based on almost 1000 cases of cochlear implants, we performed the first auditory implantation in a noise-induced hearing loss patient, and it restored the patient’s hearing [24–26]. In addition, we took part in the research and development of the first cochlear implant made in China, which conforms to the features of Chinese tone and breaks the monopoly of imported products.

2) Breakthrough in gene therapy of noise-induced hearing loss

We found that the expression of the Math1 gene increased hair cell numbers in guinea pigs. It could induce the regeneration of hair cells and improve hearing [27, 28]. The combination of DAPT (a γ-secretase inhibitor in the Notch signaling pathway) and Math1 gene expression greatly increased the number of hair cells by affecting hair cell proliferation and cilium growth [28, 29]. We further developed a new nano-gene vector, which expanded the clinical practice of gene therapy in noise-induced hearing loss patients [30].

3) Studies on stem cells make progress

Studies on stem cells indicate a promising prospect for the treatment of noise-induced hearing loss [31]. We found a method to induce the differentiation of bone marrow mesenchymal stem cells (MSCs) into hair cell-like cells [32]. Embryonic stem cells (ESCs) transplanted after drug-induced hearing loss could enter the cochlea through a hole in the scala tympani and migrate to the scala media and vestibular cisterna. They could also differentiate into myosin-VIIa-expressing hair cell-like cells [33].

## Testing the hypothesis

Through the understanding and verification of this active system, we are proposing a creative method to mimic the noise-suppression function of our hearing system. To test our hypothesis, model analyses and animal model studies are needed.

Using micro-CT scanning and three-dimensional reconstructions, a digital model of our hearing system (including the external auditory canal, middle ear and inner ear) can be rebuilt and used for acoustic analysis. Through a preliminary mathematic model analysis, we found that the energy passing from the tympanic membrane to the closed tympanic foramen of the ET is less than that to an open ET (data not shown). This suggests that the opened tympanic foramen of the ET receives part of the acoustic energy and plays a specific role in energy dissipation. Every structure in the auditory system can be analyzed and tested for its particular contribution to the process of noise reduction mentioned above.

Then, we could imitate the structure with a bionic method and apply it in the manufacture of protective devices or muffling devices. For example, a bionic ET could be created through 3D printing, and its function could be verified in animal models or volunteers with ET dysfunctions. Once proven, these structures or products could be widely used in both disease treatment and industrial manufacturing. Similar mechanisms to those mentioned above, such as the stapedial reflex, are also candidates. Products designed based on this theory will overcome the limitations of traditional reverse-wave elimination and damping absorbers.

## Implications of the hypothesis

The hearing system is a complex neurobiological feedback control system, and researchers have discovered the active cochlear amplifier, which is important in evolution. We think the hearing organ has equipped itself with a self-protection mechanism, which we summarize as a function of active noise reduction. We should realize that under different frequency or intensity conditions, different effects may be present in the same structure.

Bionic structures manufactured based on this theory would have a noise reduction function and could be used in the treatment of patients with noise reduction problems, for which there is no current medical treatment. For example, 3D–printed ETs could be implanted to improve dysfunctions in the original ETs.

On the other hand, it is very important especially for the military industry and military noise prevention. The application of this type of bionic auditory structure in weaponry or military equipment will greatly decrease military noise-induced damages.

These promotions may lead to favorable directions for the prevention and treatment of hearing loss and facilitate the development of the military industry and social health.

## Abbreviations

DAPT: γ-secretase inhibitor in the Notch signaling pathway
ESC: Embryonic stem cell
ET: Eustachian tube
MSC: Mesenchymal stem cell
